# Report of the Buffalo General Hospital

**Published:** 1866-07

**Authors:** 


### REPORT

OF THE

### Buffalo General Hospital

FOR THE YEAR 1865.

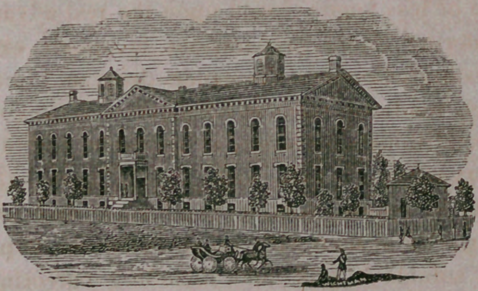

Reprint from the "Buffalo Medical and Surgical Journal."

BUFFALO:
Joseph Warren & Co., Printers, 178 Washington Street.
1866.

### THE BUFFALO GENERAL HOSPITAL.

The Buffalo General Hospital Association held their first meeting at the rooms of the Buffalo Medical Association, Nov. 21st, 1855. At that meeting the Hospital was organized, and from that date its progress has been steadily forward, until it may now be called without question, a success. Nearly all whose names were subscribed to the resolutions drawn up at the first meeting are still interested in its welfare, and many have held official connection with it up to this time.

The west wing of the proposed plan of the hospital was dedicated June 24th, 1858, with an address from the Hon. Jas. O. Putnam, a poem by Miss Matilda Stewart, remarks by the Hon. Millard Fillmore, Jesse Ketchum, Esq., Rev. Dr. Heacock, and the President of the Association, Charles E. Clarke, Esq.

The first patient was received July 15th, 1858, and from that date to Dec. 31st, last, 2,498 patients had been received, attendant with a success of treatment, which will compare favorably with that of any Hospital in the country.

Its location is superior—situate upon the highest ground in the vicinity—overlooking the city, lake and river. The Buildings were erected expressly for Hospital purposes, and in the arrangement of the wards and rooms, are admirably adapted to convenience and comfort. The larger wards are seventy feet long, twenty-seven feet wide, and fifteen feet high. By a superior ventilating apparatus, the air is continually changed, and rendered sweet and pure.

All the buildings are in excellent order—wards and rooms neatly furnished—and no hospital has better facilities for the successful treatment of patients.

It will be seen from the annexed report that this hospital has treated 864 sick and wounded soldiers. By appointment of the Surgeon-General, U. S. A., it was named a United States Army General Hospital, December 1st, 1863, and continued to treat soldiers until October 2d, last. The friends of the institution may well be proud of the reputation it earned in their treatment; and the compliments paid it by military gentlemen of eminence who, from time to time, visited it, were highly gratifying.

The expense of maintaining such an institution is of necessity large, much larger than the care of an equal number of persons in health. The receipts for board pay a portion of the expense, but there is, and always will be, a deficiency, to be made up by voluntary contribution. The hospital has no fund, as some institutions, upon which to draw, and their property being free of encumbrance, they desire to keep it so. To make up the deficiency of the last year, the trustees will call upon the citizens of Buffalo for assistance. This report will be circulated among the friends of the hospital that they may understand what we have done and what we hope to do. The trustees are convinced that when the wants of the hospital are known, the people of Buffalo will readily assist in placing this institution upon a firm basis, and maintaining it in a liberal manner.

### REPORT OF THE TREASURER

FOR THE YEAR ENDING DECEMBER 31, 1865:

RECEIPTS.

| | | | |
|---|---|---|---|
| Cash on hand at last Annual Report, | | | $2,280 59 |
| For board of Soldiers | | $15,130 50 | |
| " " Sailors | | 1,496 79 | |
| " " County Patients | | 1,736 14 | |
| " " Naval Patients | | 73 50 | |
| " " Private Patients | | 2,017 97 | |
| Total for Board | | $20,454 90 | |
| Interest and Exchange | | 290 22 | |
| Loan of A. J. Rich, Esq | | 4,000 00 | |
| Articles sold | | 545 66 | |
| DONATIONS OF— | | | |
| Brayley & Pitts | $200 00 | | |
| Mary Pitts | 300 00 | | |
| Pratt & Co | 250 00 | | |
| Rumsey & Co | 250 00 | | |
| Jewett & Root | 150 00 | | |
| Buffalo Gas Light Co. (value 1,000 bushels Coke) | 200 00 | | |
| John Warner | 150 00 | | |
| Geo. B. Wilson | 100 00 | | |
| Sidney Shepard | 100 00 | | |
| A. C. Blackburn | 50 00 | | |
| H. Utley | 50 00 | | |
| DeWitt C. Weed | 50 00 | | |
| Geo. L. Squires | 50 00 | | |
| S. T. Williams | 50 00 | | |
| J. W. Hutchinson | 50 00 | | |
| E. G. Spalding | 25 00 | | |
| F. S. Pease | 10 00 | | |
| M. L. Comstock | 10 00 | | |
| Athearn & Co | 10 00 | | |
| Cornell & Son | 25 00 | | |
| Letchworth Bros | 25 00 | | |
| C. J. Hamlin | 25 00 | | |
| Young, Lockwood & Co | 25 00 | | |
| Tweedy & Smith | 10 00 | | |
| Wm. H. Walker | 10 00 | | |
| James Adams | 5 00 | | |
| Carried forward | $2,180 00 | | |

| | | | |
|---|---|---|---|
| Brought forward | $2,180 00 | | |
| D. F. Day | 5 00 | | |
| Wm. C. Sherman | 5 00 | | |
| S. N. Tifft | 5 00 | | |
| Geo. T. Bentley | 5 00 | | |
| Wm. C. Bryant | 5 00 | | |
| Cash | 16 99 | | |
| | | $2,221 99 | |
| State of New York appropriation of 1864 | | $3,282 98 | $30,795 75 |
| Total receipts | | | $33,076 34 |

EXPENDITURES.

| | | |
|---|---|---|
| Paid note due Erie Co. Savings Bank | $4,000 00 | |
| Paid note due Jas. Brayley, Esq. | 500 00 | |
| | | $4,500 00 |
| Balance due Superintendent | | 9 90 |
| Paid audited bills, charged to the following Divisions: | | |
| Furniture | $1,633 60 | |
| Buildings and Grounds | 277 54 | |
| Meats, Fish and Poultry | 4,440 88 | |
| Vegetables and Fruit | 992 09 | |
| Butter and Eggs | 2,350 43 | |
| Groceries | 2,967 87 | |
| Lights | 491 54 | |
| Fuel | 2,425 06 | |
| Contingencies | 74 26 | |
| Horse keeping | 305 72 | |
| Salaries and Wages | 5,253 00 | |
| Taxes on Vacant Lots | 11 90 | |
| Liquors | 488 98 | |
| Milk | 1,386 55 | |
| Medicine | 777 05 | |
| Surgical Instruments | 49 21 | |
| Insurance | 110 00 | |
| Board | 147 50 | |
| Interest and Exchange | 26 80 | |
| Ice | 116 10 | |
| Stationery | 89 45 | |
| Repairs | 1,423 90 | |
| Bread and Flour | 1,580 88 | |
| | | $27,420 31 |
| Cash on hand Dec. 31st, 1865 | | 1,146 13 |
| | | $33,076 34 |

The Hospital owns, free from all encumbrance—

| | | |
|---|---|---|
| The Hospital grounds, buildings and furniture, valued at | | $50,000 00 |
| Four city lots, valued at | | 3,000 00 |
| An Erie county bond for | | 5,000 00 |
| Total assets not immediately available | | $58,000 00 |
| There is due from the State the appropriation of 1865, estimated at | $4,000 00 | |
| Due for board of sailors | 452 98 | |
| From county of Erie | 1,041 00 | |
| From individuals | 213 80 | |
| From commissioners of emigration | 17 42 | |
| Uncollected donations | 725 00 | |
| Cash on hand | 1,146 13 | |
| Available assets | | $7,596 33 |
| Total assets | | $65,596 33 |

LIABILITIES.

| | | |
|---|---|---|
| Bills and accounts | $7,426 68 | |
| Notes payable | 6,000 00 | |
| Due George Howard | 736 71 | |
| Paving tax | 680 17 | |
| Total | | $14,843 56 |
| Balance | | $50,752 77 |

Respectfully submitted,

GEO. S. WARDWELL,

Treasurer.

### REPORT OF THE SUPERINTENDENT

FOR THE YEAR ENDING DECEMBER 31, 1865.

*To the Board of Trustees of the Buffalo General Hospital :*

GENTLEMEN :—I have the honor to submit the following Report of what was accomplished by this Hospital during the past year, also a few statistics of previous years.

The first patient was received into this Hospital July 15, 1858.

| No. Received. | Soldiers. | Civilians. | Total. |
|---|---|---|---|
| In 1858 | | 42 | 42 |
| " 1859 | | 135 | 135 |
| " 1860 | | 160 | 160 |
| " 1861 | | 217 | 217 |
| " 1862 | | 253 | 253 |
| " 1863 | 81 | 237 | 318 |
| " 1864 | 611 | 208 | 819 |
| " 1865 | 172 | 382 | 554 |
| | 864 | 1634 | |

Total number received since the Hospital was opened ...... 2498
Total number of deaths during the same time, being an average of 9 per cent. ........ 227
Largest per centage of deaths was in 1863............ 16 per cent.
Smallest " " " 1864............ 3.5 "

In the summer of 1863 this Hospital, although not at that time a "Military Hospital," received nearly a hundred soldiers, belonging to New England regiments, who were on their way home from the Port Hudson campaign. Many were so exhausted they could not recover; upwards of one-half died. This will account for the large per centage of deaths in that year.

Number of Patients in Hospital January 1st, 1865:

| | | |
|---|---|---|
| Soldiers | 87 | |
| Sailors | 8 | |
| County Patients | 8 | |
| Private Patients | 3 | |
| Free | 1 | |
| | | 107 |
| Males | 102 | |
| Females | 5 | |
| | | 107 |

Number of Patients received during the year:

| | | |
|---|---|---|
| Males | 493 | |
| Females | 61 | |
| | — | 554 |

Number of Patients under treatment ........ 661

| | | | | |
|---|---|---|---|---|
| Of | these | 259 | were | Soldiers. |
| " | " | 137 | " | Sailors. |
| " | " | 129 | " | County Patients. |
| " | " | 105 | " | Paying Patients. |
| " | " | 31 | " | Free Patients. |
| Total | | 661 | | |

Number of Patients Discharged—

| | | |
|---|---|---|
| Well | 455 | |
| Relieved | 96 | |
| Not relieved | 8 | |
| Not treated and eloped | 7 | |
| Dead | 35 | |
| | — | 601 |

Remaining December 31st, 1865:

| | | |
|---|---|---|
| County Patients | 28 | |
| Paying Patients | 14 | |
| Sailors | 14 | |
| Emigrants | 2 | |
| Free | 2 | |
| | — | 60 |
| Males | 46 | |
| Females | 14 | |
| | — | 60 |

---

Proportion of deaths to number of Patients treated..5.7 per cent.

Average number of Patients for the year ........ 86

---

| | | |
|---|---|---|
| Largest number in Hospital at one time, | June 1st | 134 |
| Smallest " " " " | Nov. 14th | 44 |

---

| | | |
|---|---|---|
| Number of Medical Cases treated | 382 | |
| " Surgical " " | 279 | |
| | — | 661 |

---

Number of important surgical operations, in all but two which the patients were relieved ........ 19

---

Average number of days patients remained in the Hospital ........ 34

Nativities.

| | | |
|---|---|---|
| New York State | 364 | |
| Other States | 67 | |
| Great Britain | 49 | |
| Ireland | 63 | |
| Germany | 61 | |
| Other European countries | 31 | |
| British Provinces | 26 | |
| Total | | 661 |

Occupation.

| | | |
|---|---|---|
| Soldiers and discharged soldiers | 362 | |
| Sailors | 157 | |
| Mechanics | 20 | |
| Domestics | 41 | |
| Laborers | 37 | |
| Clerks | 6 | |
| Wives | 12 | |
| Merchants | 6 | |
| Teachers | 3 | |
| Physicians | 1 | |
| Lawyers | 1 | |
| Farmers | 6 | |
| Nurses | 7 | |
| Minors | 2 | |
| Total | | 661 |

EXPENSES.

| | | |
|---|---|---|
| Meats, Fish and Poultry | $4,902 49 | |
| Vegetables and Fruits | 1,271 43 | |
| Bread and Flour | 1,680 28 | |
| Milk | 1,493 94 | |
| Butter and Eggs | 2,225 72 | |
| Groceries | 2,916 31 | |
| Ice | 162 60 | |
| Total for Stores | | $14,652 77 |
| Stables | 500 96 | |
| Fuel | 2,533 26 | |
| Lights | 623 86 | |
| Salaries and Wages | 5,253 00 | |
| Medicines | 952 28 | |
| Carried forward | | $9,863 36 |

| | | |
|---|---|---|
| Brought forward | $9,863 36 | $14,652 77 |
| Liquors | 632 31 | |
| Contingencies | 74 47 | |
| | | $10,570 14 |
| | | $25,222 91 |
| Less amount fuel stores, &c., on hand | | 1,541 00 |
| Running Expenses | | $23,681 91 |
| Furniture | $2,101 00 | |
| Repairs | 1,904 21 | |
| Buildings and Grounds | 107 76 | |
| Stationery | 164 80 | |
| Surgical Instruments | 49 13 | |
| Taxes | 11 90 | |
| Insurance | 117 82 | |
| Interest | 26 80 | |
| Board | 412 00 | |
| | | $4,895 42 |
| Total Expenses | | $28,577 33 |

*Average Expense per Week for Maintaining Patients.*

Average Running Expenses for each Patient.... $5.28 per Week.
Average Total " " " .... 6.36 "

EARNINGS.

| | |
|---|---|
| Board of Soldiers | $13,023 25 |
| " Sailors | 1,741 62 |
| " County Patients | 2,774 42 |
| " Emigrants | 17 42 |
| " Private Patients | 2,626 23 |
| Total Earnings | $20,182 94 |
| Running Expenses | $23,681 91 |
| Deficiency | $3,498 97 |
| Total Expenditures | $28,576 33 |
| Earnings | 20,182 94 |
| Total Deficiency | $ 8,393 39 |

### TABLE

### OF QUANTITY OF PRINCIPAL STORES PURCHASED DURING THE YEAR, WITH AVERAGE COST OF EACH.

| Quantity. | Articles. | Av. Cost. |
|---|---|---|
| 27,319 | lbs. Beef | $0 12.6 |
| 1,887 | " Pork and Hams | 17.7 |
| 6,466 | " Mutton | 13.4 |
| 406 | " Veal | 14 |
| 949 | " Poultry | 18 |
| 1,846 | doz. Eggs | 28.5 |
| 2,171 | lbs. Fish | 10.3 |
| 630 | bush. Potatoes | 90 |
| 71 | " Turnips | 92 |
| 17 | " Onions | 2 10 |
| 1,279 | lbs. Squash | 4 |
| 277 | " Lard | 20 |
| 4,432 | " Butter | 39 |
| 13,972 | loaves Bread | 8 |
| 4,211 | lbs. Flour | 6 |
| 975 | " Meal | 1.2 |
| 128 | bush. Apples | 1 20 |
| 875 | lbs. Rice | 14 |
| 23 | galls. Molasses | 1 20 |
| 1,100 | lbs. Salt | 1 |
| 5,394 | galls. Milk | 27 |
| 442 | lbs. Crackers, | 11 |
| 54,000 | " Ice, (per 100) | 30 |
| 183 | galls. Vinegar | 23 |
| 179 | lbs. Coffee | 37 |
| 440 | " Tea | 1 17 |
| 4,390 | " Brown Sugar | 16.5 |
| 514 | " Refined Sugar | 23 |
| 13 | bbls. Ale | 10 00 |
| 88 | galls. Whisky | 3 33 |
| 137 | tons Coal | 9 20 |
| 1,070 | bush. Coke | 20 |
| 96 | cords Wood | 7 50 |
| 138,000 | feet Gas, (per 1,000) | 4 00 |
| 558 | bush. Charcoal | 13 |

It will be noticed that the number of patients treated has increased yearly, since the Hospital was opened. The number of civilian patients under treatment in 1865 being nearly double that of 1864.

During the last year some much needed improvements have been made, private rooms refitted, and the buildings put into thorough repair. The hospital is now well furnished for as many patients as its capacity will allow, and the *items* of furniture and repairs will be much smaller the ensuing year.

While prices of all articles have ruled very high throughout the year, the management have adhered to the policy of keeping their patients well in every respect, and while strict attention has been given to prevent waste, in any department, all purchases have been made with a view to quality, rather than cheapness. The schedule of quantities used, and average price paid for the principal articles of consumption, which will be found above, may prove of interest.

Efforts have been made to extend a knowledge of the Hospital and its benefits by a judicious system of advertising, issuing circulars, &c. The expense of so doing will be found in the list of expenditures, under the head of board, and the result is a gratifying increase in the number of our private patients.

The thanks of the institution is due to the Buffalo Water Co., for their kindness in allowing the free use of their hydrants, and to the numerous business firms and gentlemen who have so generously donated. A list of the names, with the amount donated, will be found in the Treasurer's report; also to the numerous friends who, from time to time, sent in packages of useful articles, reading matter, &c., all of which was very acceptable.

The Hospital is much indebted to the gentlemen of the medical staff for their prompt and faithful attendance, also to our former resident physician, Dr. John M. Brown, now of Westfield, who for nearly two years was always ready and efficient in the discharge of his duties; also to the present acting resident physician, whose attention to his duties is worthy of all praise.

It is the earnest desire of the officers of the Hospital to keep it a first class institution, where the sick may find a temporary home, which they can look back upon after leaving it, with pleasure, not disgust, as is often the case with those obliged to resort to the care of hospitals. And no effort will be spared on their part to maintain the reputation it has so well earned.

Respectfully submitted,

WILLIAM C. BAGLEY,

Superintendent.

Buffalo General Hospital, December 31st, 1865.

### OFFICERS

### 1866.

| | |
|---|---|
| President, - - - - - - | JAMES D. SAWYER. |
| Vice-President, - - - - | JAMES BRAYLEY. |
| Secretary and Treasurer, - - | GEO. S. WARDWELL. |

### Board of Trustees.

| | | |
|---|---|---|
| JAMES D. SAWYER, | JAMES BRAYLEY, | HENRY MARTIN, |
| CHAS. E. CLARKE, | E. P. DORR, | BRONSON C. RUMSEY, |
| ANDREW J. RICH. | GEO. S. WARDWELL. | GEO. T. BENTLEY. |

### MEDICAL AND SURGICAL STAFF.

### Board of Consultation.

| PHYSICIANS. | SURGEONS. |
|---|---|
| THOMAS F. ROCHESTER, M. D., | H. N. LOOMIS, M. D., |
| GEORGE N. BURWELL, M. D., | JOHN HAUENSTEIN, M. D., |
| P. H. STRONG, M. D., | C. C. F. GAY, M. D. |

### Board of Attendance.

| PHYSICIANS. | TERM. | SURGEONS. |
|---|---|---|
| S. F. MIXER, M. D., | July 1st to Nov. 1st. | SANDFORD EASTMAN, M. D. |
| C. C. WYCKOFF, M. D., | Nov. 1st to March 1st. | JULIUS F. MINER, M. D. |
| J. N. BROWN, M. D., | March 1st to July 1st. | J. R. LOTHROP, M. D. |

| | |
|---|---|
| Superintendent, - - - | WM. C. BAGLEY. |
| Matron, - - - - - | MRS. R. W. BAGLEY. |
| Resident Physician, - - - | E. L. SHURLEY. |

